# Electrocardiographic parameters and heart rate variability in free-ranging Jaguars (*Panthera onca*) immobilized with tiletamine–zolazepam–dexmedetomidine

**DOI:** 10.1186/s12917-026-05285-2

**Published:** 2026-01-14

**Authors:** Karina Resende Assoni, Joares Adenilson May Júnior, Amanda Sarita Cruz Aleixo, Miriam Harumi Tsunemi, Renee Laufer Amorim, Alessandra Melchert, Luiz Henrique de Araújo Machado, Maria Lucia Gomes Lourenço

**Affiliations:** 1 São Paulo State University (UNESP), School of Veterinary Medicine and Animal Science, Botucatu, São Paulo Brazil; 2 Laboratório de Protozoologia e Rickettsioses Vetoriais, Federal University of Rio Grande do Sul (UFRGS), Porto Alegre, Brazil; 3 Panthera Corporation, New York, United States; 4 São Paulo State University (UNESP), Institute of Biosciences, Botucatu, São Paulo Brazil

**Keywords:** Jaguars, Autonomous modulation, Animal welfare, Dissociative anaesthetics, Alpha 2 adrenergic agonist, Wildlife immobilization

## Abstract

Understanding the physiology and particularities of wild felids is important for improving the ability of veterinary medicine to contribute to the preservation of these species. Thus, the objective of this study was to describe the electrocardiographic parameters and heart rate variability (HRV) of wild jaguars (*Panthera onca*) captured in Brazil. The anaesthetic protocol used for chemical restraint of the animals involved the drug combination of tiletamine and zolazepam (7 mg/Kg) along with the α2 adrenergic (receptor) agonist dexmedetomidine (10 µg/Kg). The 42 electrocardiographic reports from 39 animals were categorized (sex, estimated age, and body weight) and statistically analysed; 25 females and 14 males between two and 21 years old with a mean weight of 80.96 ± 23 kg were analysed. During the examination, the mean heart rate maintained by the animals was 99 bpm, with a maximum heart rate during the examination of 122 bpm and a minimum heart rate of 93 bpm; the values were comparable with the reference values for large felids. Electrocardiographic parameters differed between the sexes (*p* < 0,05) (P wave amplitude, QT interval and ST segment) and between the estimated ages (P wave amplitude) and the body weight (P wave amplitude and S amplitude) of the animals. The HRV determined by electrocardiogram did not differ (SDNN 52.89 ± 50.37, rMSSD 59.99 ± 59.79, ApEntropy 0.47 ± 0.36, CVI 3.03 ± 1.35, CSI 1.75 ± 0.82, alpha 1 DFA 0.821 ± 0.264, alpha 2 DFA 0.689 ± 0.385); however, the effect of tiletamine–zolazepam in combination with an anaesthetic agent such as dexmedetomidine (an α2 adrenergic receptor) has never been described. Since this anaesthetic protocol has currently been used effectively in wild felids, the description of electrocardiographic parameters and HRV in free-ranging jaguars seems to be particularly important for future studies of the autonomic tone in the sedation of this species.

**Supplementary Information:**

The online version contains supplementary material available at 10.1186/s12917-026-05285-2.

## Introduction

Many species are threatened with extinction, and conservation protocols are of paramount importance for maintaining them. Cardiac evaluation is an integral part of a clinical analysis of animals of different species; however, for wild animals, this evaluation requires special planning and logistics, considering the physiological behaviour and anaesthetic management of the animals. For this reason, studies of the physiological parameters of healthy animals are essential in the routine care by professionals in the area for the performance of safe anaesthesia and determination of accurate diagnoses.

In this context, one of the available assessment tools is the electrocardiogram (ECG), which, by recording the electrical potentials produced in the heart, is able to identify arrhythmias and suggest the enlargement of heart chambers and electrolyte imbalances [1]. Regarding the difference between the species of felids, the available literature indicates that the electrocardiographic parameters of wild felids are similar to those of domestic felines [2, 3].

Some of the differences noted were as follows: compared with domestic felines (*Felis catus*), cheetahs (*Acinonyx jubatus*) had a longer duration and less variation in the PR interval, greater amplitude of R waves, greater amplitude and duration of QRS and a greater QT interval. Such alterations can be explained by the level of anaesthetic sedation during the procedure (ketamine and xylazine), variation in the vagal tone, and species-specific differences, such as the greater size of the heart muscle and greater distance between the heart and the electrodes [2, 4].

Another available assessment method is a heart rate variability (HRV) analysis, which should be understood as an adaptation mechanism between stressors [5]. Changes in its parameters can be associated with pathological conditions of different origins [6, 7], including situations of physiological or behavioural stress in domestic cats [8]. In spectral analyses of HRV, the level of influence of the sympathetic and parasympathetic systems on heart rate can be observed [9], as these systems are regulated by proprioceptive receptors, chemoreceptors and baroreceptors that send information to the brainstem [10].

Although no studies addressing HRV in big cats have been reported in the literature, a study of domestic felines under anaesthesia concluded that the choice of anaesthetic agent influences HRV parameters and that dexmedetomidine can initially increase heart rate because of the alpha agonist effect and subsequently reduce it because of vasoconstriction and the baroreflex response [11].

Thus, during the evaluation of cardiac parameters in anaesthetized wild animals, the effects of anaesthesia and how they alter cardiovascular function must be considered. In the present study, an α2 adrenergic agonist (dexmedetomidine, DEX), dissociative anaesthetics (ketamine and tiletamine) and benzodiazepines (zolazepam) were used for chemical restraint and anaesthesia of the animals. Dissociative anaesthetics such as ketamine (which is widely used for initial chemical restraint) and tiletamine increase cardiac output by stimulating the myocardium and increasing cardiac work and thus oxygen consumption [12]. In addition, they promote a decrease in coronary vascular resistance in an attempt to improve the blood and oxygen supply.

In terms of the pharmacodynamic properties of DEX, it shows high affinity and selectivity for a2-adrenoceptors; this pharmacological characteristic determines a typical biphasic haemodynamic response. After infusion, DEX induces sympatholytic effects, such as a lower mean arterial blood pressure (MAP) and heart rate (HR), through the activation of presynaptic a2-adrenoceptors in the central nervous system. In addition, DEX induces initial vasoconstrictive effects followed by vasodilation through the activation of a2-adrenoceptors in endothelial cells. The vasoconstrictive effects of DEX are attributable to the activation of a2-adrenoceptors in vascular smooth muscle, resulting in a clinical increase in MAP and a decrease in HR [12].

Despite its sympathomimetic effect with positive inotropic and chronotropic effects, ketamine causes few changes in blood pressure and myocardial electrical conductivity. On the other hand, when used alone, tiletamine causes premature ventricular depolarizations and can lead to seizures in wild cats [13]. Therefore, ketamine and tiletamine should preferably be used in combination with benzodiazepines such as zolazepam and midazolam, which, despite their minimal sedative effects on domestic mammals, are used for their myorelaxant effects and potentiation of the effects of anaesthetic agents, promoting greater cardiovascular stability at appropriate doses [13].

The tiletamine/zolazepam combination promotes prolonged containment in carnivores, but tigers and leopards are sensitive to tiletamine, which causes apathy, hyperopia, and neurological signs during the postanaesthetic period [14]. Finally, α2 adrenergic agonists, such as DEX, produce potent sedative, analgesic, and myorelaxant effects but lead to vasoconstriction and hypertension, with consequent reflex bradycardia due to the increase in vagal tone through the baroreceptors; thus, a decrease in cardiac output occurs, which then causes a decrease in peripheral vascular resistance, leading to hypotension, persistent bradycardia, and even second-degree atrioventricular blocks [15].

The general objective of the present study was to describe the electrocardiographic parameters and heart rate variability (HRV) of clinically healthy jaguars (*Panthera sp*.) under dissociative anaesthesia and to analyse their possible electrocardiographic patterns. In addition, parameters that influence HRV were evaluated, and both objectives aimed to enrich the currently available literature to assist future diagnostic processes in the routine veterinary care of wild felids. Additionally, this study will help researchers distinguish more explicitly between the lack of reference ECG values for large felids and differences in cardiovascular responses between anaesthetic agents. Beyond the clinical evaluation and monitoring of breathing rate, heart rate, body temperature and reflexes, ECG is an additional monitoring tool to further evaluate the effectiveness of anaesthesia and the health status of the animal being studied.

## Materials and methods

### Animal restraint and anaesthesia protocol

Electrocardiographic parameters (ECGs) and HRV were measured under anaesthesia in wild jaguars (*Panthera onca*) present in a conservation project (Southern Pantanal of Mato Grosso MS, Brazil; latitude: −20°39 × 55.5´´555; longitude: − 56°43 × 16.6´´666) [16, 17]. Between 2011 and 2020, 39 jaguars—14 males and 25 females—were captured under SISBio 61,844, 52,734 and 30,053.

Automatic trigger cameras monitored the jaguar capture sites and then snare-type traps were placed at points determined by an analysis perform by the cameras of the increases in the frequency and passage of the target animals.

Physical restraint was performed using the snare technique equipped with a VHF transmitter and monitored at a distance every hour from 4 p.m. to 9 a.m. Once the change in the signal of a specific transmitter was detected, the team moved to the trap site. The animals were captured using snare traps. All the traps had a VHF alarm system, which was checked every hour. When the signal changed, the team moved to the location to performed the procedure.

#### Anaesthesia

Anaesthesia was induced using an anaesthetic dart fired from a rifle. Once the dart was applied, the time between anaesthesia induction and measurement was approximately 20 min. The darts used were from the Daninject^®^ brand, nylon models and needles with collar, Denmark^®^ (Olgas Allé 4 DK-6000 Kolding Denmark).

The captured species was visually confirmed, and untargeted animals were released without the need for an anaesthetic protocol. Jaguars were inspected for identification and weight estimation for the preparation of dart with anaesthetic drugs. Using a CO_2_ pressure rifle, the animal was sedated with a combination of tiletamine hydrochloride and zolazepam hydrochloride (Telazol, 7 mg/kg; Fort Dodge, Campinas-SP, Brazil) and with DEX (Dexdomitor, 10 µg/kg; Fort Dodge, Campinas-SP, Brazil). The injection sites for anesthetic darts are generally the gluteal and scapular muscle region, due to the greater volume of muscle mass there. The felines were observed until they presented with ataxia, lateral recumbency, and total loss of consciousness. After capture, the animals were weighed on a digital scale (Cabelas^®^, brand, digital model, USA) with a capacity of up to 150 kg to confirm the estimated weight.

#### Division of animals and clinical examination

The animals were also divided into three subgroups according to body weight: the group weighing 38–69 Kg, whose estimated mean age was 77 ± 32.32 months; the group weighing 70–86 Kg, whose estimated age was 130 ± 63.87 months; and the group weighing between 92 and 127 Kg, whose estimated age was 114 ± 33.87 months.

The ages of the animals were estimated based on the period of monitoring using camera traps, which allow for the individual recognition and tracking of each animal by observing the rosettes on their coats. After capture, the body characteristics and dental changes (tooth wear and gum changes) of the animals were assessed.

Immediately after anaesthesia, the animal was removed from the trap, and then a general clinical evaluation was conducted, mainly to assess external changes that could be evaluated, such as those in the teeth and skin. Then, a clinical examination of the patient’s rectal temperature, heart and respiratory rates, and staining of mucous membranes were performed. We included only animals that were healthy upon clinical examination. After the clinical examination of the previous parameters, an electrocardiographic evaluation was performed. All the jaguars were equipped with a GPS radio collar (Lotek Wireless, Inc., Canada) [18]. The examinations were performed 15 min after the administration of the anaesthetics.

### Electrocardiogram

Electrocardiographic reports from 39 animals—25 females and 14 males—were analysed. The animals were positioned in the right lateral recumbency position for five minutes of digital electrocardiographic recording using a portable ECG (InPulse Animal Health^®^, Florianópolis, Santa Catarina). The electrocardiogram began approximately 30 min after sedation.

**Fig. 1:**
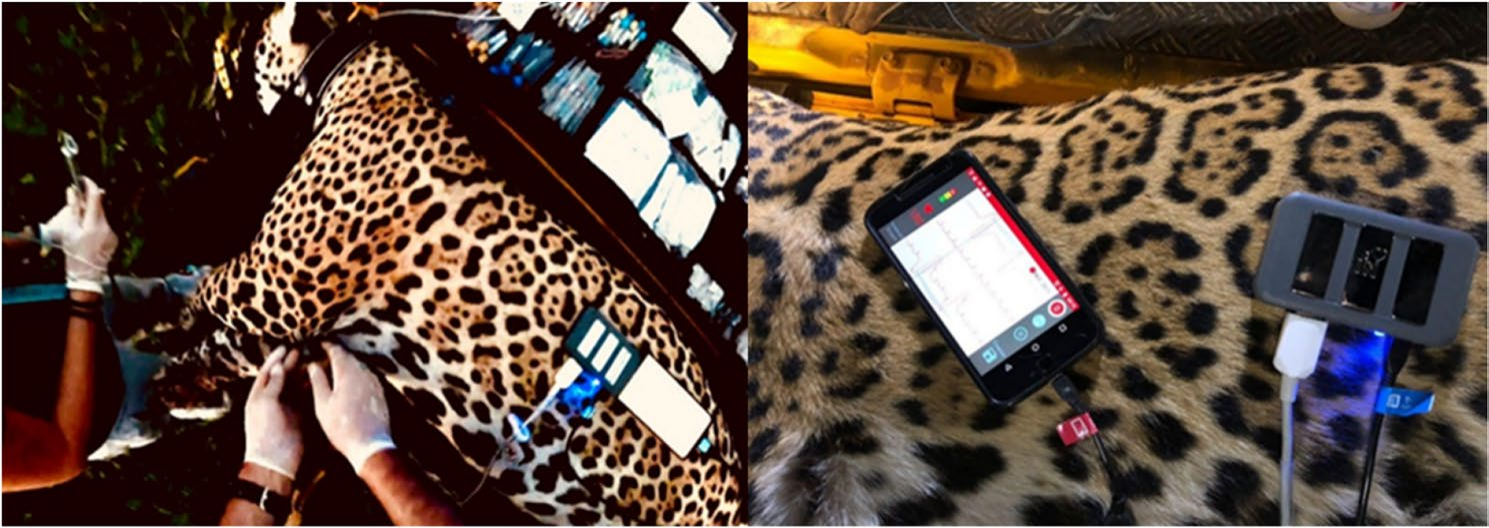
An electrocardiographic examination was performed on jaguars anaesthetized with tiletamine/zolazepam and dexmedetomidine

Bipolar leads (I, II, and III) and augmented unipolar leads (aVR, aVF, and aVL) were recorded according to the standard limb electrode placement using four electrodes placed directly on the animal’s skin, two electrodes placed in the femur–tibio–patellar joint (patellar ligament) and two electrodes placed in the humero–radio–ulnar region (olecranon). The data were subsequently transferred to the cloud using the inCardio^®^ program, where the traces were evaluated and later interpreted to prepare the report (Fig. 1).

The following electrocardiographic parameters were evaluated during the study: maximum, mean and minimum heart rate (bpm); heart rhythm; durations and amplitudes of the P, R, S and T waves; PR and QT interval durations; ST segment; T wave polarity; and the QRS and P electrical axes evaluated using lead II.

###  Heart rate variability

Heart rate variability was assessed by the linear method in the time domain, considering the standard deviation of all RR intervals (SDNN), which represents sympathetic activity, and the root mean square of the successive differences (rMSSD), which represents parasympathetic activity, as determined from the analysis of adjacent RR intervals.

For the nonlinear methods, also called spectral methods, the evaluation indices used were approximate entropy (ApEN) (which measures irregularity or randomness in a set of data such that smaller values reflect greater regularity), cardiovagal index (CVI) (which is a measure of parasympathetic activity and is calculated from the Poincaré plot as the logarithm of the product of the longitudinal axis and the transverse axis), the cardiosympathetic index (CSI) (which is a measure of sympathetic activity and is calculated from the Poincaré plot as the ratio of the longitudinal axis to the transverse axis), and the detrended fluctuation analysis (DFA) (which quantifies the fractal-like correlation properties of a time series and reveals short-range (DF α1) and long-range (DFα2) correlations within a series and sympathetic and parasympathetic percentages).

After the exams were performed, a radio collar was placed on the animals, which is important for continued monitoring after their release. Other evaluations of the animal included biometrics (obtaining measurements that allow possible future comparisons of the same animal) and weighing. At the end of the collection of samples and clinical examinations, the animal was allowed to recover and was released in the same place where it was captured.

### Statistical analysis

For statistical analysis, the animals were divided by sex, estimated age (less than and greater than 120 months) and weight.

The Shapiro‒Wilk normality test was used to assess the data distribution and to compare the means/medians between sexes and ages.

For the analysis of parametric data, Student’s t test was used, and for the analysis of nonparametric data, the Mann‒Whitney test was used. For comparisons of means or medians between weight ranges, we used ANOVA and the Kruskal‒Wallis test, respectively.

For the correlation analysis, if the P value was greater than 0.05, Pearson’s correlation test was used; for variables that did not pass the normality test, Spearman’s correlation test was used. The Bonferroni correction was performed to control type I error (false positives) when multiple comparisons were performed simultaneously.

Statistical analyses were conducted using the R Core Team (2024). Statistical significance was set at 95% (*P* < 0.05).

## Results

The estimated age was 109 ± 51.58 months and differed significantly (*p* = 0.047) between jaguar females (102 ± 57.68 months) and males (123 ± 33.99 months). The overall body weight was 81 ± 23.68 kg, and females weighed less (70 ± 14.48 kg) than males (104 ± 17.73 kg) (*p* = 0.000).

### Electrocardiographic parameters

#### Heart rate, cardiac rhythm, morphology and axis

Approximately 20 min after the capture and sedation of the animals, the electrocardiogram was performed for 3 to 5 min according to the methodology (Fig. 1).

The mean heart rate was 99 ± 22.7 bpm (range: 93 ± 22.8 to 123 ± 74.7 bpm), with no significant difference between sexes ( Table 1) (*p* = 0.18) or estimated age (Table 2) (*p* = 0.74) or body weight subgroups (*p* = 0.57) (Table 3).

**Table 1:** The means and standard deviations (medians) of electrocardiography data obtained from the DII lead in free-ranging Jaguars anaesthetized with tiletamine/zolazepam and Dexmedetomidine that were stratified according to sex

Parameters	Sex^a^ Mean and standard deviation (median)	
	**Female** (*n* = 30)	**Male** (*n* = 14)
HR min (bpm)	91.50 ± 20.83 (94)	96.71 ± 27.07 (86.50)
HR med (bpm)	96.6 ± 19.04 (99)	106.42 ± 28.83 (97)
HR max (bpm)	111.23 ± 48.55 (102)	148.84 ± 112.90 (104)
P wave (ms)	57.26 ± 14.95 (60)	65.57 ± 31.93 (60)
P wave (mV)	0.04 ± 0.03 (0.04)^a^	0.09 ± 0.06 (0.08)^a^
PR interval (ms)	121 ± 29 (123)	123 ± 41 (134)
QRS complex (ms)	100.53 ± 28.54 (95)	104.57 ± 37.39 (99)
QT interval (ms)	290 ± 54 (300)^a^	238 ± 75 (234)^a^
R wave (mV)	0.44 ± 0.27 (0.4)	0.40 ± 0.18 (0.38)
ST segment (ms)	111.53 ± 45.87^a^ (120)	74.28 ± 48.66^a^ (58)
ST segment (mV)	0.02 ± 0.02 (0.02)	0.04 ± 0.045 (0.02)
T wave (mV)	0.09 ± 0.08 (0.09)	0.10 ± 0.13 (0.045)
QRS electrical axis (°)	69 ± 36 (77.06)	83 ± 14 (78.14)
P electrical axis (°)	40 ± 88 (73.89)	46 ± 94 (76.53)

^a^ Statistically significant differences between sexes: the Mann‒Whitney test was used to assess the P wave amplitude (p=0.003) and QT interval (p=0.016); T test was used to assess the ST segment duration (p=0.018)

**Table 2:** The means and standard deviations (medians) of electrocardiography data obtained from the DII lead in free-ranging Jaguars anaesthetized with tiletamine/zolazepam and dexmedetomidine that were stratified according to age

Parameters	Age^a^ Mean and standard deviation (median)	
	**< 120 months** (*n* = 24)	**> 120 months** (*n* = 15)
HR min (bpm)	90.66 ± 19.32 (91)	95.60 ± 28.17 (97)
HR med (bpm)	98.54 ± 18.55 (96.5)	101.06 ± 28.90 (101)
HR max (bpm)	128.04 ± 89.87 (100)	119.73 ± 60.97 (103)
P wave (ms)	56.08 ± 13.93 (59)	64.93 ± 31.41 (62)
P wave (mV)	0.04 ± 0.03 (0.05)	0.07 ± 0.06 (0.06)
PR interval (ms)	120 ± 33 (123)	125 ± 34 (130)
QRS complex (ms)	104.83 ± 28.38 (96)	94.80 ± 36.06 (96)
QT interval (ms)	280 ± 58 (295)	254 ± 83 (268)
R wave (mV)	0.41 ± 0.23 (0.39)	0.44 ± 0.27 (0.4)
ST segment (ms)	102.83 ± 44.86 (112)	92.40 ± 58.53 (94)
ST segment (mV)	0.01 ± 0.01 (0.015) ^a^	0.05 ± 0.04 (0.04)
T wave (mV)	0.07 ± 0.06 (0.06)	0.11 ± 0.15 (0.05)
QRS electrical axis (°)	77 ± 31 (82.63)	68 ± 36 (74.24)
P electrical axis (°)	42 ± 83 (73.54)	35 ± 104 (79.31)

^a^Statistically significant difference between ages: the Mann‒Whitney test was used to analyse the ST segment amplitude (*p* = 0.013)

**Table 3:** The means and standard deviations (medians) of electrocardiography data obtained from the DII lead in free-ranging Jaguars anaesthetized with tiletamine/zolazepam and Dexmedetomidine that were stratified according to weight

Parameters	Weight^*^ Mean and standard deviation (median)		
	38–69 Kg (*n* = 15)	70–86,5 Kg (*n* = 16)	92–127 Kg (*n* = 13)
HR min (bpm)	92.33 ± 21.97 (90)	94.75 ± 25.27 (99.5)	92.15 ± 22.03 (84)
HR med (bpm)	95.93 ± 21 ± 85 (93)	102.68 ± 24.20 (103)	100.61 ± 23.01 (91)
HR max (bpm)	99.66 ± 22.39 (97)	127.68 ± 63.44 (108)	144.50 ± 117.82 (100)
P wave (ms)	56.0 ± 17.21 (52)	59.62 ± 30.06 (60)	64.76 ± 13.45 (64)
P wave (mV)	0.04 ± 0.03 (0.04) ^*^	0.07 ± 0.06 (0.05)	0.07 ± 0,02 (0,08) ^*^
PR interval (ms)	120 ± 37 (132)	118 ± 31 (121)	128 ± 31 (136)
QRS complex (ms)	100.26 ± 14.49 (96)	100.12 ± 42.22 (93)	105.69 ± 31.63 (98)
QT interval (ms)	292 ± 45 (296)	269 ± 86 (304)	257 ± 55 (272)
R wave (mV)	0.41 ± 0.24 (0.38)	0.50 ± 0.29 (0.49)	0.37 ± 0.19 (0.31)
ST segment (ms)	115.20 ± 39.53 (126)	96.25 ± 48.91 (110)	86.00 ± 58.49 (84)
ST segment (mV)	0.01 ± 0.01 (0.015) ^*^	0.04 ± 0.03 (0.03) ^*^	0.03 ± 0.04 (0.20)
T wave (mV)	0.09 ± 0.09 (0.09)	0.13 ± 0.13 (0.11)	0.05 ± 0.05 (0.05)
QRS electrical axis (°)	78 ± 18 (77.86)	71 ± 36 (78.10)	72 ± 37 (75.71)
P electrical axis (°)	65 ± 79 (78.86)	14 ± 96 (61.29)	49 ± 90 (78.97)

^*^Statistically significant differences between weight groups: the Kruskal–Wallis test was used to assess the P wave amplitude in the 38–69 Kg and 92–127 Kg groups (*p* = 0.04) and the ST segment amplitude in the 38–69 Kg and 70–86,5 Kg groups (*p* = 0.008)

**Fig. 2:**
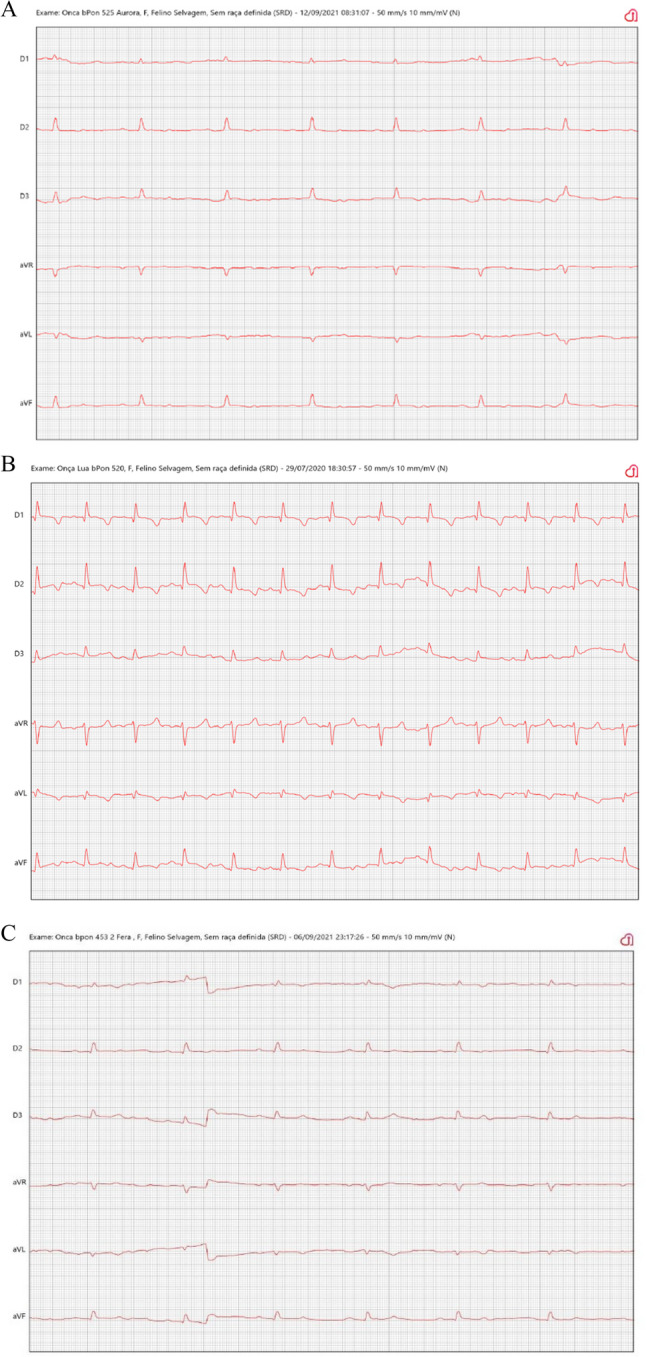
**A** Sinus rhythm (HR = 82 bpm); (**B**) sinus tachycardia (HR = 140 bpm); and (**C**) sinus arrhythmia (HR = 75 bpm) in female jaguars

In terms of the heart rhythms detected during the study, 85.71% of jaguars had a normal sinus rhythm (Fig. 2 A), 9.52% had sinus tachycardia (Fig. 2B) and 4.76% had sinus arrhythmia with a migratory pacemaker (Fig. 2 C). Non physiological arrhythmias or cardiac electrical conduction disorders were observed during the examination.

An examination of the morphology of the electrocardiographic waves revealed that the P wave was positive in all leads of the frontal plane and that the Q and S waves were small and narrow when present. In most of the traces, the amplitude of the ST segment (0.03 ± 0.03 mV) was normal, except for a 12-year-old male with a tachycardia sinus and a weight of 81 kg (Table 2 and 3).

The QRS complex was positive at the DI, DII, DIII and aVF leads and negative at the aVR and aVL. The mean amplitude of the R wave was 0.43 ± 0.25 mV. The polarity of the T wave was positive in most of the electrocardiographic traces (75.56%) and negative in 24.44%.

The mean cardiac electrical axis of the QRS complex in the frontal plane was 74 ± 31°, and that of the P wave was 42 ± 89°.

**Fig. 3:**
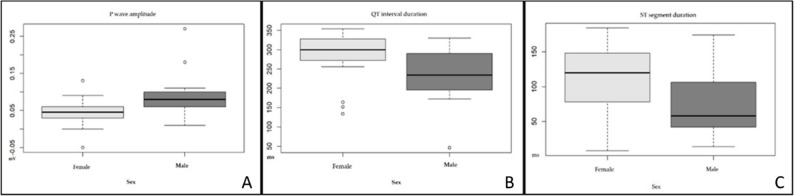
Significant differences in the parameters (P wave—mV, QT interval—ms, ST segment duration—ms) between male and female wild jaguars immobilized with tiletamine–zolazepam–dexmedetomidine (Mann‒Whitney test for the P wave amplitude and QT interval; T test for the ST segment duration)

In terms of the effects of sex on ECG parameters, significant differences were observed between males and females in the following parameters: the QT interval (*p* = 0.01), which was higher in females (290 ± 54 ms) than in males (238 ± 75 ms); the ST segment (*p* = 0.01), which was also higher in females (111.53 ± 45.87 ms) than in males (74 ± 48.66 ms); and the P wave amplitude (*p* = 0.003), which was lower in females (0.04 ± 0.03 mV) than in males (0.09 ± 0.06) (Table 1; Fig. 3).

**Fig. 4:**
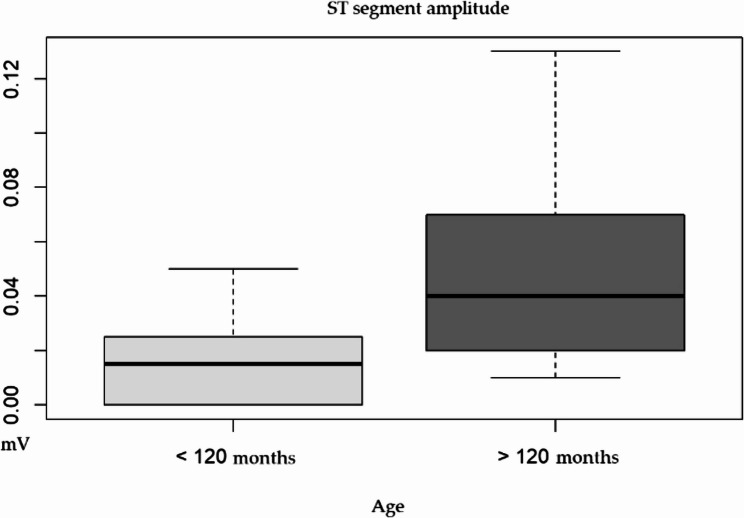
Significant differences in the parameter between age groups (<120 vs. > 120 months) of wild jaguars immobilized with tiletamine–zolazepam–dexmedetomidine (T test for the ST segment amplitude)

With respect to age groups, the ST segment amplitude (*p* = 0.001) was significantly different. Animals with an estimated age of less than 120 months presented a lower value (0.01 ± 0.01 mV) than those over 120 months (0.05 ± 0.04 mV) (Table 1; Fig. 4).

**Fig. 5:**
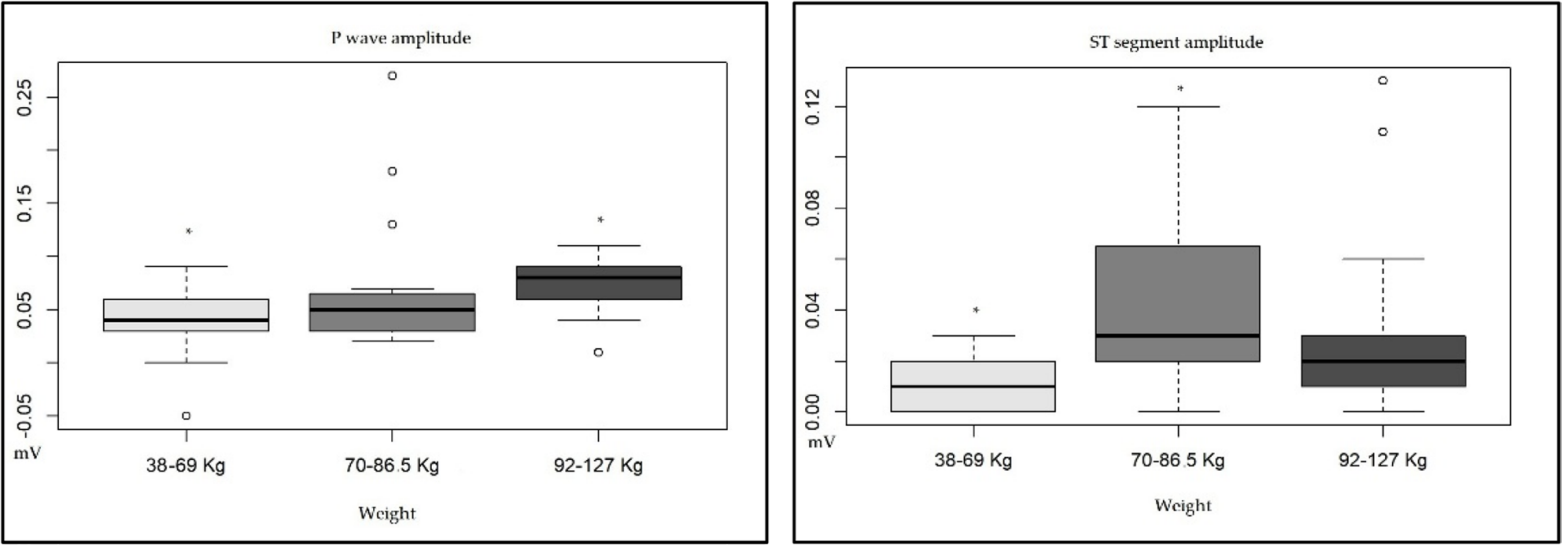
Graphs showing significant differences (p < 0.05) in the electrocardiographic parameters of the P wave amplitude and ST segment amplitude obtained from the DII lead in wild jaguars immobilized with tiletamine–zolazepam–dexmedetomidine

With respect to the weight of the jaguars, significant differences in the P wave amplitude (*p* = 0.04) between the 38–69 kg (0.04 ± 0.03 mV) and 92–127 kg weight groups and in the S wave amplitude (*p* = 0.008) between the 38–69 kg (0.01 ± 0.01 mV) and 92–127 kg weight groups. The weight of this group ranged from 70 to 86 kg (0.04 ± 0.03 mV) (Table 1; Fig. 5).

We did not observe any adverse effects of the anaesthesia protocol used on any of the animals in the present study.

### Heart rate variability parameters

Regarding the parameters of HRV (Table 4), no significant differences were observed between sexes (females and males), between the estimated age groups (< 120 kg or > 120 kg) or between the different body weight ranges (38–69 kg, 70–86.5 kg and 92–127 kg). However, significant correlations were observed between the minimum, mean and maximum heart rates and the mean NN (RR intervals) and the alpha 2 DFA index. A significant negative correlation (*r*=−0.76; *p* = 0.0000) was identified between the minimum heart rate and the mean NN and a weak positive correlation was observed between the minimum heart rate and the alpha 2 DFA (*r* = 0.01; *p* = 0.388). The mean heart rate was also negatively correlated with the mean NN (*r*=−0.91; *p* = 0.000) and positively correlated with the alpha 2 DFA (*r* = 0.30; *p* = 0.05). The maximum HR was negatively correlated only with the mean NN (*r*=−0.79; *p* = 0.000).

**Table 4:** The heart rate variability parameters of Jaguars are presented as the means ± standard deviations and (medians)

Parameters*	Values
HR min	93.15 ± 22.81 (92.50)
HR med	99.77 ± 22.72 (99)
HR max	122.6 ± 74.66 (103)
Mean NN	580.54 ± 175.17 (587.79)
SDNN	52.89 ± 50.37 (28.56)
rMSSD	59.99 ± 59.79 (30.57)
ApEntropy	0.47 ± 0.36 (0.42)
CVI	3.03 ± 1.35 (2.91)
CSI	1.75 ± 0.82 (1.54)
Alpha 1 DFA	0.821 ± 0.264 (0.79)
Alpha 2 DFA	0.689 ± 0.385 (0.65)
Sympathetic percentage	37.72 ± 17.77 (33.46)
Parasympathetic percentage	62.27 ± 17.77 (66.54)

*HR min, med, max minimum, median and maximum heart rate, *ApEntropy* Approximate entropy, *CVI* Cardiovagal index, *CSI* Cardiosympathetic index, *α1 and α2 DFA* Detrended fluctuation analysis

Table 5 shows a comparison of electrocardiographic data from wild felids analysed in various studies.

**Table 5:** Comparisons between electrocardiographic parameters of different felid species, studies and anaesthetic protocols

Parameters	The present study Panthera onca	Santilli [1] Domestic cats	Oda, 2007 [22] Herpailurus yagouaroundi	Larson et al., 2008 [4]		Oda, 2009 [3] Leopardus tigrinus	Oliveira, 2016 [21] Puma concolor	Kirnew et al., 2025 [20] Panthera onca
				Panthera leo	Panthera tigris			
HR med (bpm)	99.77 ± 22.72	140–220	91 ± 17	64 ± 12	81.53 ± 15.19	107 ± 17	94.82 ± 13.25	72 ± 18
P wave (ms)	59.90 ± 21.79	< 35	30 ± 10	60 ± 10	50 ± 9	50 ± 70	75.9 ± 7.12	49 ± 9
P wave (mV)	0.06 ± 0.04	< 0.2	0.03 ± 0.10	0.09 ± 0.04	0.09 ± 0.02	0.13 ± 0.05	0.091 ± 0.008	0.12 ± 0.03
PR interval (ms)	122 ± 33	50–90	90 ± 10	160 ± 24	167 ± 40	100 ± 80	121.6 ± 24.06	133 ± 22
QRS complex (ms)	101.81 ± 31.24	< 40	50 ± 10	70 ± 10	50 ± 10	50 ± 10	58.54 ± 7.85	54 ± 8
QT interval (ms)	273 ± 66	160–220	240 ± 20	300 ± 20	225 ± 28	230 ± 30	255.45 ± 19.30	301 ± 33
R wave (mV)	0.43 ± 0.25	< 0.9	1.08 ± 0.45	0.93 ± 0.34	0.92 ± 0.26	1.45 ± 0.6	0.55 ± 0.29	0.67 ± 0.23
ST segment (ms)	99.68 ± 49.43	normal	normal	normal	normal	normal		246 ± 27
ST segment (mV)	0.03 ± 0.03	normal	normal	normal	normal	normal		0.04 ± 0.03
S wave (mV)	0.03 ± 0.03	-	-	absent	maximum of 0.1	-		0.06 ± 0.13
T wave (mV)	0.10 ± 0.11	maximum + 0.3	positive	25.9 +; 22.3-; 51.8 +/-	93.3–7.7	positive		0.34 ± 0.17
QRS electrical axis (°)	74 ± 31	0°-+160°	-		60–90	60–90	-	82 ± 13
P electrical axis (°)	42 ± 89	0°-+90°	-			-	-	81 ± 14

*In the present study, tiletamine, zolazepam and dexmedetomidine were used; in [1], unanaesthetised domestic cats were studied; in [19], xylazine and ketamine were used; in [4], xylazine and ketamine were used; in [3], xylazine and ketamine were administered; in [20], sevoflurane or isoflurane was administered; and in [21], medetomidine and ketamine were used

## Discussion

### Anaesthetic protocol

Chemical immobilization of wild animals is a form of veterinary anaesthesia that is necessary when animals cannot be safely handled in an appropriate restraint area with restraint tools. Several combinations of drugs have been used to immobilize wild felids, and the choice of drugs is often dictated by factors such as the availability cost of the drug, size and species of the felid during the procedure, the purpose and the health status of the animal [22, 23], setting (wild vs. captive), induction time, amount and route of administration, and duration and pain of the procedure.

We did not observe differences in HR with respect to sex, age or weight; however, we observed that female animals older than 120 months and with lower weights presented higher HRs, which may be due to the small number of animals in each group; however, sex, age and body condition (fat) must be considered when restraint methods are used. We did not observe any adverse effects with the sedation protocol used in any of the animals in the present study, which is inherent to different age groups and sexes, demonstrating that this protocol is safe in jaguars.

Tiletamine is a dissociative anaesthetic marketed in combination with zolazepam (TZ). This combination results in the rapid and safe immobilization of species with minimal cardiovascular and respiratory depression. The addition of zolazepam aims to potentiate the effects of tiletamine, promote central‑acting myorelaxation, and minimize undesirable the side effects typical of dissociative anaesthesia. The disadvantages of the TZ are the lack of visceral analgesia and the absence of a specific antagonist. Tiletamine and zolazepam are typically combined with an alpha-2 agonist to reduce these side effects. Medetomidine (M) is a highly selective alpha-2 agonist. Compared with TZ alone, the combination of medetomidine–tiletamine–zolazepam (MTZ) produces deep anaesthesia characterized by greater analgesia and muscle relaxation [24].

Medetomidine is a mixture of levomedetomidine and DEX. Dexmedetomidine is considered the only active isomer and induces weaker sedation and stronger antinociception than medetomidine does. However, the addition of medetomidine to tiletamine–zolazepam is believed to cause hypertension, bradycardia and arrhythmias [25], which were not observed in the animals in the present study, and we also used alpha-2 agonists.

The administration of α2 adrenergic receptor agonists is associated with adverse effects on the cardiovascular system, such as a biphasic blood pressure response (hypertension followed by normal or hypotension) with reflex bradycardia and decreased cardiac output, increased systemic vascular resistance index and central venous pressure and bradyarrhythmia [26]. In the present study, despite the use of anaesthetics combined with dexmedetomidine, the animals did not present bradycardia, with a mean HR of approximately 99 bpm 20 min after drug administration, a finding that resembles that reported by Souza et al. (2018) [27], who documented an HR value of 94 bpm (± 12) in jaguars anaesthetized with tiletamine, zolazepam and dexmedetomidine.

###  Electrocardiographic parameters

Threatened with extinction in Brazil, the jaguar is the largest predator in Latin America and plays an important role in the ecosystem. In recent years, studies have revealed important aspects of the movement ecology of the jaguar. The social system of the jaguar is based on territoriality, with adults maintaining individual territories. The use of space by animals is directly influenced by environmental factors such as the availability of resources and the presence of other animals [18].

Spatial overlap has been described between males and females and male–female pairs [28]. Since intervention in this species requires chemical containment methods, electrocardiographic evaluation becomes essential for making the procedure safer. Opportunistic capture (handlers take advantage of pre-existing situations, rather than actively luring the animal into a trap at a predetermined location and time - e.g., using carcasses as bait) may have occurred in studies with this design, which may also have been influenced by the clinical condition of the animals; however, this process did not occur in the present study.

Larson et al. (2008) [4] reported mean HR values of 64 bpm for lion (*Panthera leo)* and 81 bpm for tiger (*Panthera tigris*). The HR of the animals in the present study was higher, which may have been due to the protocol used to restrain the animals and stress capture, since the authors of the previous study used ketamine and xylazine, providing evidence of the bradycardic effect of xylazine. In terms of the amplitude of the P wave, we detected lower and positive values and no differences in the P wave between the sexes, similar to the findings of Larsson et al. (2008). In our study, the predominant rhythm was sinus rhythm.

In their study of jaguars sedated with medetomidine and ketamine, Kirnew et al. [21] reported higher P wave amplitudes in females than in males, differing from the results of our study in which males presented higher P wave amplitude values; females in our study presented longer QT intervals and ST segment durations, since they presented lower HR values. Weight is a factor to be considered when assessing the amplitude of the P wave, as animals with a higher weight presented higher values, as well as the amplitude of the ST segment. Older animals are heavier, so the effects of age and weight need to be teased out.

Age can influence the amplitude of the ST segment, with older animals having a greater amplitude. We did not observe differences in the P wave duration, perhaps because of the number of animals in the present study, but we noted that larger animals presented longer durations. A longer P wave duration may be related to atrial overload and/or interatrial conduction delay.

Kirney et al. [21] reported an increase in the durations of the PR and QT intervals in jaguars compared with domestic cats in 2025. In the present study, we obtained lower values ​​of the PR and QT interval than those in the study by Kirnew et al. [21],; however, it the animals that were included in the study are wild. The lifestyles of jaguars and other species must be considered when electrocardiographic parameters and HRV indices are analysed. We believe that the fact that the jaguars in our study are wild and no sedentary and have a natural diet that is more similar to that of the species when they live in the wild may have influenced the electrocardiographic parameters. Animals with inadequate diets may have greater overweight and body fat, influencing the duration of electrocardiographic waves.

The choice of protocol used to restrain animals when evaluating cardiovascular parameters should be made with caution. In their study, Oliveira et al. (2016) [20] aimed to evaluate the electrocardiographic parameters in pumas anaesthetized with isoflurane and sevoflurane and obtained the predominant sinus rhythm; despite the adoption of a different protocol in the present study, the majority of jaguars also presented sinus rhythm. Regarding the average HR, the animals in our study presented higher values ​​(99 bpm) than did the animals in the study by Oliveira et al. (2016) [20] (94 bpm), illustrating the effect of the anaesthetic protocol on HR, where tiletamine can cause an increase in heart rate due to its sympathomimetic action, while zolazepam has sedative, hypnotic, muscle relaxant and anticonvulsant properties, with minimal effects on the cardiovascular and respiratory systems.

With respect to the duration of the QT interval, we observed higher values ​​in our study than in the studies by Oda et al. (2007) [19] of *Herpailurus yagouaroundi* (Jaguarundi), Oda (2009) [3] of *Leopardus tigrinus* (Gato-do-Mato), and Oliveira (2016) [20] of *Puma concolor* (Onça Parda). In addition to the anaesthetic protocol used, species differences may play a role in electrocardiographic parameters and should be considered for analysis.

Regarding the amplitude of the T wave, most of the animals in the present study presented positive values. With respect to the amplitude of the R wave, we observed a positive polarity similar to that reported by the aforementioned authors but with lower amplitudes (0.43 and 0.68, respectively); this difference is expected, which may be to the detriment of the greater body mass in jaguars.

### Heart rate variability parameters

The variation in RR intervals is influenced by the activity of the autonomic nervous system (ANS). HRV refers to the short-term variation in the time interval between consecutive heartbeats, which depends on the interaction between the parasympathetic and sympathetic components in cardiovascular control. The considerable variation and complexity of HRV provides advantages in terms of the body’s adaptability to address a wide range of challenges. The analysis of HRV reflects the ANS regulation of the cardiac cycle [29]. In their study, Xueyuan et al. (2021) [30] compared cardiopulmonary parameters after the administration of DEX, tiletamine and zolazepam to Siberian tigers and detected lower heart rate values ​​20 min after drug administration (± 84 bpm).

Living conditions should also be considered when the parameters are analysed since the animals in the present study are wild animals. The environment, type of diet and physical condition may have influenced the results and were not evaluated in the present study, but they also reflect reliable electrocardiographic parameters of the animals. Future studies are needed to evaluate the behaviour of electrocardiographic parameters and HRV by comparing wild and captive jaguars.

#### Linear indices

Negative correlations were observed between the minimum, mean and maximum heart rate values and the HRV index NN or mean RR (mean RR interval of all recordings). These results are expected since the higher the HR is, the shorter the RR interval. Despite the lack of correlation between the other HRV indices, the animals in the present study presented higher values for the rMSSD index (root mean square of the sum of the squares of the differences between adjacent RR intervals), indicating vagal activity, even in the presence of the anaesthetic effect of dexmedetomidine. Other factors, such as the activity level, environmental stress, and body temperature, should be considered when interpreting HRV indices.

Several factors influence HR and cause variation between two consecutive heartbeats. Cardiac output is regulated by the central nervous system through neural and endocrine pathways [31]. Caramalac et al. (2020) [32] administered medetomidine with tiletamine–zolazepam to *Puma concolor* and observed significant reductions in heart and respiratory rates, which can be attributed to the activity of presynaptic α−2 adrenergic receptors that lead to the inhibition of norepinephrine release with consequent bradycardia. In the present study, when we evaluated the HRV indices, a parasympathetic predominance was noted, which may be to the detriment of the aforementioned α−2 adrenergic effects. But, our findings establish baseline ECG and HRV parameters for field immobilization of large felids.”

#### Nonlinear indices

Regarding nonlinear HRV indices, the present study initially aimed to describe their behaviours in jaguars subjected to the anaesthetic protocol and was performed at shorter recording intervals. However, future studies are needed to evaluate nonlinear indices to compare wild and captive animals, as well as their use in animals with diseases, and to analyse how nonlinear HRV indices behave in healthy and unhealthy animals, since studies using nonlinear HRV methods are scarce in jaguars. The nonlinear DFA (detrended fluctuation analysis) index determines the correlation between stationary or nonstationary time series. This method calculates indices (α coefficients) according to the similarity in a set of time scales to detect cardiac abnormalities where low α1 coefficients and high alpha 2 coefficients indicate abnormalities in the dynamic system that defines the HRV [33].

DFA is a useful technique for determining fractal-scale properties and long-range correlations in noisy and nonstationary time series and has been applied to the evaluation of DNA sequences, HR dynamics, neuron spiking, human gait, and meteorological and earthquake signals. Fractal properties are an emergent property of system dynamics, and certain pathologies can disrupt its dynamics, resulting in the alteration of its fractal properties [34].

We found that the values ​​of the nonlinear HRV indices α1 and α2 in ounces (0.821 and 0.689, respectively) were similar to those in the study by Zacarias et al. (2014) [35] (1.1 and 0.75, respectively) designed to evaluate the behaviour of foetal heart rate in cardiotocographic examinations of healthy human foetuses. Despite the disparity in comparisons between human foetuses and jaguars, the similarity of values ​​indicates that the ANS activity in the animals in the present study is preserved, and further studies are needed to standardize nonlinear HRV indices in jaguars.

The animals in this study were wild, and we believe that diet may influence the analysis of HRV. Future studies are needed to compare HRVs in wild and captive jaguars, since the influence of diet on health can occur over decades, and biomarkers that help identify aspects of nutrition that have a positive or negative influence are needed. Compared with wild animals, captive animals tend to have greater modifications in their diet, as well as greater space restrictions. In humans, the HRV can be used to indicate the potential health benefits of foods. Reduced HRV is associated with the development of numerous conditions, such as diabetes, cardiovascular disease, inflammation, obesity, and psychiatric disorders.

For the CVI (cardiovascular index related to parasympathetic activity) and CSI (cardiosympathetic index related to parasympathetic and sympathetic activity), we found values ​​of 3.03 and 1.75, respectively. We observed similar values ​​to those in studies described by Pacheco et al. (2019) in dogs (CVI of 4.025 and CSI of 1.10). Future studies aiming to evaluate nonlinear HRV indices in jaguars are necessary and may contribute to describing the behaviour of ANS activity in this species. The present study is applicable as a reference for the method, since the objective was to perform descriptive analyses of these parameters.

The nonlinear HRV approximate entropy (ApEntropy) index describes the predictability or randomness of physical systems that change over time; the higher the entropy value is, the more complex the process. Regarding approximate entropy (-ApEn), the more complex (chaotic) the value is and the more regular and predictable the series is, the lower the -ApEn value.

In a study by Correa et al. (2010) [36] of humans that evaluated HRV and pulmonary infections after myocardial revascularization, the authors obtained values ​​less than or equal to 0.4802 that were associated with pulmonary infections during the postoperative period of myocardial revascularization. Approximate entropy decreases with the loss of homeostasis or chaos; namely, it approaches linear behaviour. In the present study, we obtained values for the approximate entropy (0.47) ​​close to those in the study by Correa et al. (2010) [36]; however, the anaesthetic protocol may have influenced these values ​​since it alters the HRV, and no reference values ​​in ounces are available for this index, necessitating future studies for standardization and evaluating the influence of anaesthetic protocols on nonlinear HRV indices.

With respect to linear HRV indices in the time domain, Kirnew et al. (2025) [21] reported values ​​for an SDNN of 68 ms in jaguars. In our study, we observed lower values ​​(52 ms), the value of the HRV index rMSSD for the animals in our study was 59.99, and in the study by Kirnew [21], it was 117.00, which may be due to the anaesthetic protocol adopted.

Studies have reported sex differences in autonomic regulation, with women having higher HRs than men under various conditions. However, while one might believe that a higher HR would automatically result in a lower HRV, women tend to display a higher baseline HRV than men even if they have higher HRs [37]. Although several studies of humans have revealed the influence of sex on HRV parameters, in the present study, no differences were observed between sexes for some parameters, but its influence must be considered in the analysis.

HRV can be used as a noninvasive tool to assess prognostic factors, since systemic diseases can affect ANS activity [38]. Spontaneous neoplasms are important causes of death in captive wild felines and constitute up to 51% of all postmortem diagnoses. Specifically, in jaguars, neoplasms include mast cell tumours, haemangiosarcoma, lymphoma, exocrine pancreatic carcinoma and mesothelioma [39]. Thus, the results of the present study emphasize the importance of electrocardiographic evaluation and standardization in jaguars, as obtaining HRV indices, when altered, may indicate the presence of systemic diseases characterized by a loss of HRV.

The two main pathways through which psychological stress affects the body are the hypothalamic–pituitary–adrenal (HPA) axis and the sympathetic nervous system (SNS). The SNS promotes the sympathetic response to stress, commonly referred to as a fight or flight response, by withdrawing the inhibitory effect. A series of changes follow, including the release of noradrenaline from the locus coeruleus. During the stress response, the HPA axis triggers a series of endocrine changes, beginning with the release of corticotropin-releasing hormone from the hypothalamus. Stress is associated with variations in autonomic activity that disrupt homeostatic processes. Moreover, stasis, which is a lack of endogenous variability in neurally mediated peripheral systems (HR), is a sign of severe physiological distress [40].

This study has several limitations. Differences in the anaesthetic protocol, recording environment, and equipment used for interspecies comparisons were not controlled. No reference values are available for approximate entropy in the species for an effective interpretation of our data. We did not observe significant effects according to the electrocardiogram variables and clinical parameters; this may be a limitation of the study. However, in this study, we also aimed to illustrate descriptive parameters in ounces. Perhaps a new study comparing doses and cardiovascular effects would be interesting.

The type of diet and physical condition may have influenced the results and were not evaluated in the present study. Fight-or-flight responses were not assessed, and no electrocardiographic parameters or HRV indices were used. The moments prior to capture may stimulate fight-or-flight responses in animals, but the influence of stress was not assessed in this study, which is a limitation. Additionally, in the present study, we did not correlate or evaluate blood pressure or the respiratory rate after the sedation protocol was employed or the influence of stress on these parameters, which are highlighted as limitations of the present study.

## Conclusions

Tiletamine–zolazepam–dexmedetomidine combinations can be used to immobilize free-ranging jaguars, and no adverse effects were observed. ECG and HRV measurements could be conducted opportunistically during the immobilization of free-ranging jaguars for collaring.

Preliminary results for ECG parameters and HRV were reported for the first time, and further studies are needed to determine/evaluate the effects of immobilizing drugs, stress, and condition.

## Supplementary Information

Supplementary Material 1.

Supplementary Material 2.

## Data Availability

Data and materials can be requested from the corresponding author Maria Lucia Gome Lourenço.

## Abbreviations

α1 and α2: Alpha 1 and 2 detrended fluctuation analysis
DFA: Detrended fluctuation analysis
Ap Ent: Approximate entropy
aVF: Augmented unipolar limb lead for the left leg
]aVL: Augmented unipolar limb lead for the left arm
aVR: Augmented unipolar limb lead for the right arm
bpm: Beats per minute
CSI: Cardiosympathetic index
CVI: Cardiovagal index
HR: Heart rate
P: P wave
PR: PR interval
QRS: QRS complex
QT: QT segment
NN (RR): RR intervals
rMSSD: Square root of the mean of the square of the differences between adjacent RR intervals
SDANN: Standard deviation of the means of the RR intervals obtained every five minutes
SDNN: Standard deviation of all RR intervals
ST: ST segment
T: T wave
