# Supplementary material for: Electrocardiographic parameters and heart rate variability in free-ranging Jaguars (Panthera onca) immobilized with tiletamine–zolazepam–dexmedetomidine
Source: BMC Vet Res. 2026 Jan 14;22:137. doi: 10.1186/s12917-026-05285-2 (PMC12947326; doi:10.1186/s12917-026-05285-2)
Supplement: Supplementary file 1 — Supplementary Material 1. [file 12917_2026_5285_MOESM1_ESM.pdf]

CERTIFICADO

Certificamos que a proposta intitulada Avaliação do perfil eletrocardiográfico e variabilidade da frequência cardíaca em onças (Panthera onca) sob anestesia dissociativa, registrada com o nº 000.098, sob a responsabilidade do (a)Prof(a).Dr(a) MARIA LUCIA GOMES LOURENÇO - que envolve a produção, manutenção ou utilização de animais pertencentes ao filo Chordata, subfilo Vertebrata (exceto humanos), para fins de pesquisa científica (ou ensino) - encontra-se de acordo com os preceitos da Lei nº 11.794, de 8 de outubro de 2008, do Decreto nº 6.899, de 15 de julho de 2009, e com as normas editadas pelo Conselho Nacional de Controle de Experimentação Animal - Concea, e foi aprovada pela Comissão de Ética no Uso de Animais da Faculdade de Medicina Veterinária e Zootecnia, em reunião de 17/06/2024.

Finalidade	Pesquisa Científica
Vigência da Autorização	01/08/2024 a 23/08/2025
Espécie/linhagem/raça	-
Número de animais	-
Sexo	-
Origem	-

Fábio Sossai Possebon

Fábio Sossai Possebon
Presidente da CEUA
Faculdade de Medicina Veterinária e Zootecnia
Botucatu
