# Supplementary material for: Electrocardiographic parameters and heart rate variability in free-ranging Jaguars (Panthera onca) immobilized with tiletamine–zolazepam–dexmedetomidine
Source: BMC Vet Res. 2026 Jan 14;22:137. doi: 10.1186/s12917-026-05285-2 (PMC12947326; doi:10.1186/s12917-026-05285-2)
Supplement: Supplementary file 2 — Supplementary Material 2. [file 12917_2026_5285_MOESM2_ESM.pdf]

AJE®

Editing Certificate

This document certifies that the manuscript

Electrocardiographic parameters and heart rate variability in free-ranging Jaguars (Panthera onca) immobilized with Tiletamine-zolazepam-dexmedetomidine

prepared by the authors

Karina Resende Assoni, Joares Adenilson May-Júnior, Amanda Sarita Cruz Aleixo, Mirian Harumi Tsunemi , Renee Laufer Amorim, Alessandra Melchert, Luiz Henrique de Araújo Machado, Maria Lucia Gomes Lourenço*

was edited for proper English language, grammar, punctuation, spelling, and overall style by one or more of the highly qualified English speaking editors at AJE.

This certificate was issued on **September 4, 2025** and may be verified on the AJE website using the verification code **749B-296C-9839-3C6D-063P**.

AJE EDITING CERTIFIED

Neither the research content nor the authors' intentions were altered in any way during the editing process. Documents receiving this certification should be English-ready for publication; however, the author has the ability to accept or reject our suggestions and changes. To verify the final AJE edited version, please visit our verification page at aje.com/certificate. If you have any questions or concerns about this edited document, please contact AJE at support@aje.com.

AJE provides a range of editing, translation, and manuscript services for researchers and publishers around the world.
For more information about our company, services, and partner discounts, please visit aje.com.
